# Acaricidal Activity of Tea Tree and Lemon Oil Nanoemulsions against *Rhipicephalus annulatus*

**DOI:** 10.3390/pathogens11121506

**Published:** 2022-12-09

**Authors:** Samar M. Ibrahium, Ahmed A. Wahba, Ahmed A. Farghali, Abdel-Azeem S. Abdel-Baki, Shaimaa A. A. Mohamed, Saleh Al-Quraishy, Ahmed O. Hassan, Shawky M. Aboelhadid

**Affiliations:** 1 Department of Parasitology, Animal Health Research Institute, Fayum Branch 16101, Egypt; 2 Department of Parasitology, Animal Health Research Institute, Dokki Branch 12611, Egypt; 3 Materials Science and Nanotechnology Department, Faculty of Postgraduate Studies for Advanced Sciences, Beni-Suef University, Beni-Suef 62511, Egypt; 4 Zoology Department, Faculty of Science, Beni-Suef University, Beni-Suef 62511, Egypt; 5 Health Administration, Egyptian Ministry of Health, Beni-Suef 62511, Egypt; 6 Zoology Department, College of Science, King Saud University, Riyadh 12372, Saudi Arabia; 7 Department of Medicine, Washington University School of Medicine, St. Louis, MO 63110, USA; 8 Parasitology Department, Faculty of Veterinary Medicine, Beni-Suef University, Beni-Suef 62511, Egypt

**Keywords:** *Melaleuca alternifolia*, *Citrus limon*, essential oil, tick, resistant

## Abstract

Tick infestation is a serious problem in many countries since it has an impact on the health of animals used for food production and pets, and frequently affects humans. Therefore, the present study aimed to investigate the acaricidal effects of nanoemulsions of essential oils of *Melaleuca alternifolia* (tea tree, TT) and *Citrus limon* (lemon oil, CL) against the different stages (adult, eggs, and larvae) of deltamethrin-resistant *Rhipicephalus annulatus* ticks. Three forms of these oils were tested: pure oils, nanoemulsions, and a binary combination. Tea tree and lemon oil nanoemulsions were prepared, and their properties were assessed using a zeta droplet size measurement and a UV-Vis spectrophotometer. The results showed that TT and CL exhibited higher adulticidal effects in their pure forms than in their nanoemulsion forms, as demonstrated by the lower concentrations required to achieve LC_50_ (2.05 and 1.26%, vs. 12.8 and 11.4%, respectively) and LC_90_ (4.01% and 2.62%, vs. 20.8 and 19.9%, respectively). Significant larvicidal activity was induced by the TTCL combination, and LC_50_ was reached at a lower concentration (0.79%) than that required for the pure and nanoemulsion forms. The use of pure CL oil was found to have the most effective ovicidal effects. In conclusion, pure TT and CL have potent acaricidal effects against phenotypically resistant *R. annulatus* isolates. It is interesting that the activity levels of TT and CL EOs’ binary and nanoemulsion forms were lower than those of their individual pure forms.

## 1. Introduction

*Rhipicephalus annulatus* is an obligate blood-feeding ectoparasite of cattle that is widely distributed in tropical and subtropical regions [1]. Tick infestations of cattle can harm hides and reduce production. In addition, they have the ability to transmit pathogens, including *Babesia* species and *Anaplasma marginale* [2].

Chemical acaricides are frequently used to control ticks. Pyrethroid acaricides are commonly used to control tick populations. There are serious environmental and public health issues associated with its extensive use. Deltamethrin, the most common pyrethroid, can bind to sodium channels and cause hyperexcitability in neurons and tick death [3,4]. Deltamethrin has an adverse effect on humans, animals, and the environment [5]. Due to its overuse, the *R. annulatus* tick has evolved phenotypic and genotypic resistance to deltamethrin [1,6]. It is, therefore, necessary to search for alternative products or strategies for tick control. Essential oils represent prospective substitutes to the acaricides now in use, since various studies have shown that several plants have toxicological effects on ticks [7]. The current use of essential oils is safe for humans, animals, and the environment, and the development of resistance is uncommon [8,9]. The mechanisms of action of EOs include many target locations, such as cell wall disintegration, membrane protein obliteration, and increased cell wall permeability [10].

*Melaleuca alterniolia* (*tea tree* oil) (TT) has an acaricidal effect on nymphs of *Ixodes ricinus* [11]. TT and its nanostructured form cause a mortality rate of 70%, and a 100% reduction in the egg laying and hatchability phases of female *Rhipicephalus microplus* ticks [12]. The application of *Citrus limon* (Lemon oil) (CL) was proven to induce a mortality rate of 98.68% in the mealy bug *Dysmicoccus brevipes*, with the LC_50_ and LC_95_ attained at concentrations of 0.72% and 2.91%, respectively [13]. Additionally, CL was used in rabbits to treat mange, the most severe ectoparasitic disease caused by infestation by the mite *Sarcoptes scabiei* with a 100% mortality rate in mites and complete eradication of the disease’s clinical manifestations [14]. Additionally, CL showed significant acaricidal activity against engorged *Hyalomma dromedarii* females [15]. 

The low solubility in the aqueous phase, high volatility, and low long-term stability are limiting factors for the use of EOs as natural acaricides. A promising solution is the development of essential oil nanoemulsions, which are prepared by the mixing of two immiscible liquids (oil and water) to form a single phase by means of an emulsifying agent, also known as a surfactant. The resultant nanoemulsion typically has droplet sizes between 20 and 200 nm [16]. Recently, it was found that nanocapsules and nanoemulsions containing cinnamon oil have significant activity against engorged *R. (B.) microplus* females [17,18]. Additionally, Boito et al. [19] reported that *tea tree* nanoemulsions interfere with the reproduction of *R.* (*B.*) *microplus*. Ibrahium et al. [20] found that geranium nanoemulsions were much more effective against *R. annulatus* than its pure form. 

In this study, we aimed to assess the effectiveness of TT and CL nanoemulsions compared with their pure forms and binary combination against the deltamethrin phenotypic-resistant tick *Rhipicephalus annulatus*.

## 2. Materials and Methods

### 2.1. Ethical Approval

These experiments were conducted in accordance with the ethics of the Faculty of Veterinary Medicine, Beni-Suef University, Egypt, under approval number (021-172).

### 2.2. Source of Compounds of the Study 

*M. alternifolia* (*tea tree* oil) (TT) and *C. limon* (CL) were purchased from a commercial source (Trust Scientific for Natural Products, Cairo, Egypt). The oils were dissolved in 70% ethanol at rates of 10, 5, 2.5, 1.25, and 0.625% (volume/volume). Binary mixtures from these oils were added at a rate of (1:1) for all five concentrations. This step was performed to investigate the synergistic/antagonistic activity between essential oil binary mixtures, and the synergistic factor (SF) was calculated according to Suwannayod et al. [21].

$$\mathrm{SF}=\frac{\mathrm{LC}50\;\mathrm{for}\;\mathrm{the}\;\mathrm{oil}\;\mathrm{alone}\;}{\mathrm{LC}50\;\mathrm{for}\;\mathrm{the}\;\mathrm{combination}}$$

SF > 1, indicates synergism; SF < 1, indicates antagonism.

### 2.3. GC-MS of TT and CL Oil

The GC-MS analysis was carried out using a gas chromatography–mass spectrometry instrument (TRACE GC Ultra Gas Chromatographs (THERMO Scientific Corp., Santa Clara, CA, USA). The chemical constituents of the essential oils were identified using AMDIS software (www.amdis.net) as well as by assessing their retention indices (relative to n-alkanes C8-C22), matching their mass spectra to authentic standards and consulting the Wiley Spectral Library collection and the NSIT Library database (Nawah Scientific Educational Research Center, Cairo, Egypt). 

### 2.4. Nanoemulsion Preparation of TT and CL

TT and CL nanoemulsions were formulated using three components in accordance with Nirmala et al. [22]: TT or CL oil, Tween 80, and distilled water. The nanoemulsion formulation process was a two-step process, initiated with preparations of oil/water macroemulsions by blending TT or CL oil, surfactant (Tween 80), and water, at a specific concentration of one oil to two T80 using a magnetic stirrer at a speed of 500 rpm for 10 min. The TT or CL oil concentration was 10%. Then, the emulsions were prepared by forming the respective nanoemulsions using an ultrasonicator with a 750 W input power processor (Branson Probe sonicator-Advanced model, 20 kHz, Swedesboro, NJ, USA). Each concentration was subjected to different sonication time periods of 5, 10, and 15 min, respectively. The turbulence produced by the high-energy shockwaves disrupted the droplets. The mild heat generated was regulated by positioning the sample in a container filled with ice.

### 2.5. Characterization of Nanoemulsion

The absorbance of the emulsions was measured at 270–345 nm with the help of a UV-visible spectrophotometer (UV-2600, Shimadz, Japan). The zeta potential, droplet size distribution (d, nm) (analysis by volume) and polydispersity index (PDI) of the nanoemulsions were measured by a zeta sizer apparatus (dynamic light scattering technique) (Nano-ZS90, Malvern, UK). Prior to the experiment, all samples were diluted to 10% with deionized water in order to reduce the effects caused by multiple scattering effects.

### 2.6. Acaricide Activity of the Prepared Materials 

#### 2.6.1. Preparation of Ticks 

Female *R. annulatus* ticks engorged by blood were collected from naturally infested cattle from El-Fayoum Governorate, Egypt (85 km southwest of Cairo). The ticks were collected from cattle with a history of recurrent tick infestation that had not received any treatment for four weeks. The collected ticks were transported to the Parasitology Laboratory, Faculty of Veterinary Medicine, Beni-Suef University. They were identified in accordance with Estrada-Peña [23]. The ticks were divided into Petri dishes containing 10 ticks each, for the adult immersion bioassay. A second group of collected ticks was incubated in a BOD incubator and allowed to oviposit. Eggs were collected, mixed, weighed, and then allocated to test tubes in 50 mg lots for the ovicidal activity test. Some of the allotted eggs were then incubated until hatching. The hatched larvae were used for the larval packet test.

#### 2.6.2. Adult Immersion Test (AIT) 

The prepared concentrations of EOs (TT and CL), nanoemulsions (TTN and CLN), and their combinations (TTCL) were tested at 5 different concentrations against adult engorged female ticks. The ticks were immersed in petri dishes containing 10 mL samples of the tested solutions for 2 min and dried with filter paper. Then, they were incubated in a BOD incubator (26–28 °C and 80% relative humidity) [24]. For each concentration, 5 replicates (10 ticks for each replicate) were used. The negative control was ticks treated with 70% ethyl alcohol. The positive controls were: one treated with 2 mL/L deltamethrin (Butox®, CAS: 52918-63-5) (pyrethroid insecticide as sodium channel modulator), and the other one treated with 0.5 mL/L Phoxim 50% (Sebacil®, CAS: 14816-18-3) (organophosphate insecticide as an acetylcholinesterase inhibitor). The number of dead adult ticks were counted after 14 days, and the egg production index (EPI) was reported for live ticks [25]. The egg production index (EPI) was calculated as follows: EPI = weight of egg mass/initial weight of engorged females × 100.

#### 2.6.3. Ovicidal Activity 

Fifty micrograms of tick eggs were placed on filter paper that was 4 × 7 cm in size. Then, 1 mL of the tested concentration was applied to the eggs in plastic mesh for 2 min. Then, the eggs were dried on filter paper and incubated in glass tubes closed with cotton [24]. The control eggs were treated with 70% ethanol, 50% phoxim, and 5% deltamethrin. The eggs from the treated groups were incubated in a BOD incubator until hatching. For each concentration, three replicates were used. 

#### 2.6.4. Larvicidal Activity 

A modified larval packet technique (LPT) described by [26,27] was used. Using a paint brush, approximately one hundred larvae were placed on the center of 7 × 7 cm filter papers. Then, 100 µL samples of the test solutions were added. The papers were then enclosed to form packets. Control group samples were treated with ethanol (70%). For each concentration, 5 replicates were used. The treated packets were investigated after 24 h to record mortality rates.

#### 2.6.5. Statistical Analysis

The results of the different treatments were statistically analyzed using IBM SPSS for Windows, v.22 (IBM, Armonk, NY, USA). The difference between treatments was compared using ANOVA, and subsequent Duncan’s tests were used to estimate the differences between means (α = 0.05). The lethal concentrations and 50%, and 90% mortalities were calculated with SPSS v.22. 

## 3. Results

### 3.1. GC-Mas Analysis of Tea Tree and Citrus Limon Oils

Terpinene (52.24%), dihydro-α-terpineol (5.97), and diterpene (2.87%) are the main constituents of TT (Table 1). Meanwhile, (L)-alpha-terpineol (18.32%), alpha-terpinol (13.43%), trans-4-thujanol (9.64%), α- terpinolene (5.81%), citral propylene glycol acetal (5.73%), geranial propylene glycol acetal (4.00%), and α-terpineol acetate (3.60%) are the major components of CL (Table 1). 

### 3.2. Characterization of Oil Nanoemulsions

**Figure 1:**
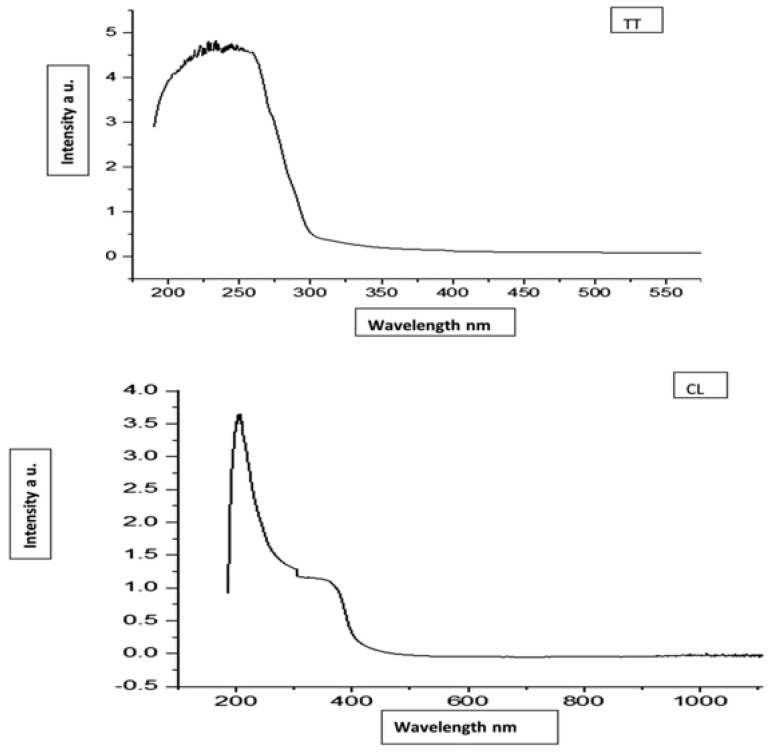
UV-vis spectrophotometer absorbance of *tea tree* (TT) and limon (CL) oils.

**Figure 2:**
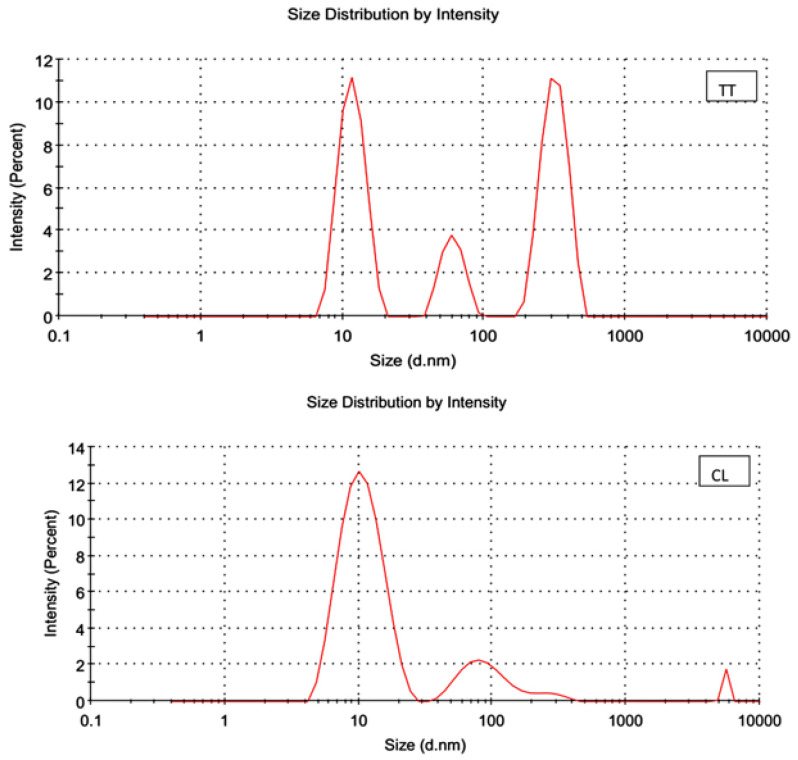
Size distribution of *tea tree* (TT) and lemon oils (CL) by intensity using Zeta apparatus.

**Figure 3:**
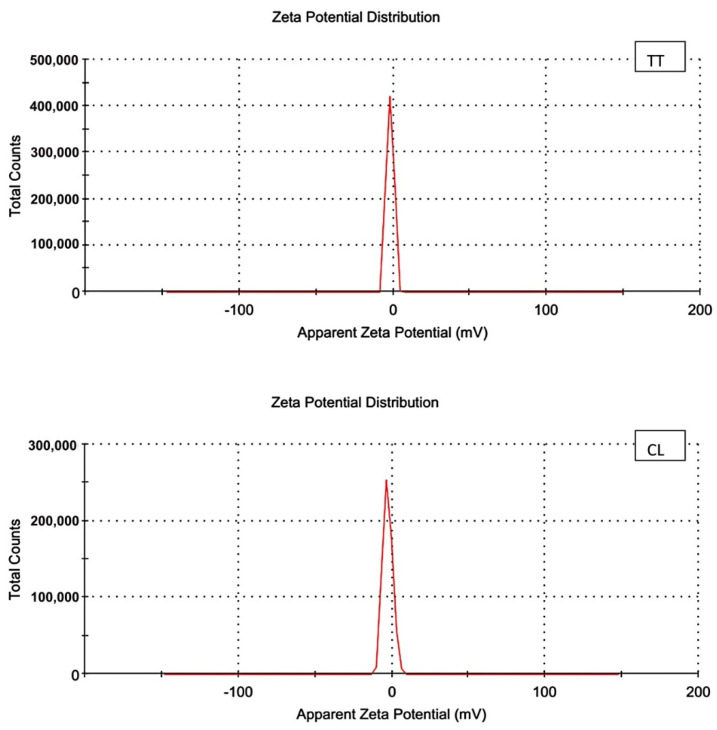
Zeta potential distribution of *tea tree* (TT) and lemon (CL) oils by Zeta apparatus.

The UV-Vis spectrophotometer detected the absorbance peaks of CLN and TTN at 345 nm and 270 nm, respectively (Figure 1). Zeta potential measurements revealed negative charges on the outer surface of TT N (−2.02 mV) and CLN (−2.30 mV) and the hydrodynamic particle sizes of TTN and CLN with mean droplet sizes of approximately 188.4 d.nm and 50.66 d.nm and PDI values of 0.527 and 0.171, respectively (Figure 2 and Figure 3). The low PDI values reflect the homogeneous size distribution of the EO droplets (Table 2).

### 3.3. Adulticidal Activity of Oils against R. Annulatus Tick

The adulticidal activities of TT and CL against *R annulatus* ticks were significant (*p* ˂ 0.05), especially at concentrations of 5 and 10%, where they induced mortality rates of 96.60 and 100%, respectively (Table 3). No deaths were reported in the deltamethrin (2 µL/mL) treated ticks. Meanwhile, 100% mortality was observed in the Phoxim-treated ticks (Table 3).

**Figure 4:**
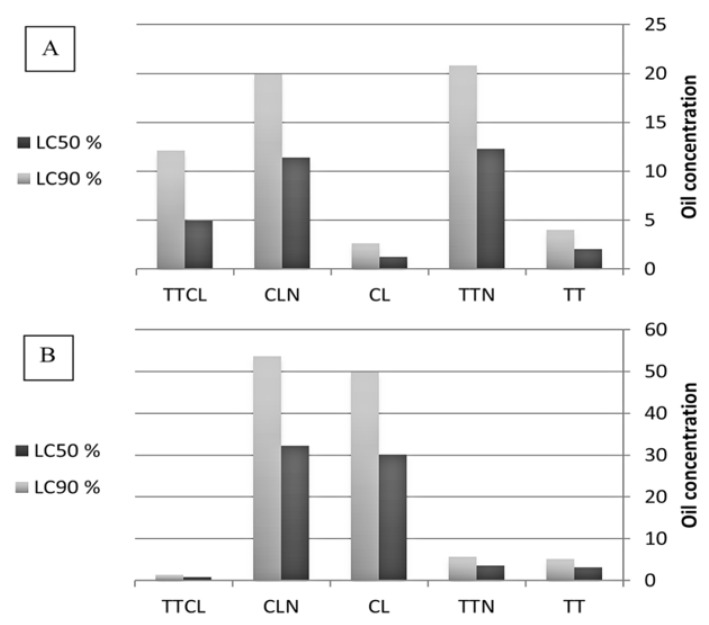
LC_50_ and LC _90_ of *tea tree* and *citrus lemon* oils against (**A**) adult of *R. annulatus*, and (**B**) larvae of *R. annulatus*.

The lowest dosages of TT and CL exhibited lower egg production indices (EPI) than control ticks (treated with ethyl alcohol 70%), whereas TTN and CLN were associated with similar EPI values to the control ticks (Table 4). The LC_50_ values for the pure TT and CL oils were 2.05 and 1.26%, respectively, whereas they were 12.3 and 11.4%, respectively, for the nanoemulsion forms (Figure 4). Furthermore, the corresponding LC_90_ values for TT and CL were 4.00 and 2.62%, but they were 20.8% and 19.9% for TTN and CLN.

### 3.4. Ovicidal Activity of EO Formulations

CL and CLN significantly inhibited egg hatching (*p* ≤ 0.05), achieving inhibition rates of 90.66 and 69.00%, respectively, at the highest concentration (10%). At the same time, neither TT nor TTN had significant effects on the egg hatching percentage at any of the tested concentrations, and the treated eggs hatched normally (Table 5). The eggs treated with Phoxim 50% showed significant inhibition of egg hatching, achieving an inhibition percentage of 82.66%, whereas deltamethrin-treated eggs were found to have no effect on the hatching rate, achieving the same hatchability percentage as the untreated control eggs (Table 5).

### 3.5. Larvicidal Activity of EO Formulations

TT and TTN only exhibited significant larval mortality at high concentrations, as the mortality rate reached 100% and 92.3% at a concentration of 10% and 100%, and 83.3% at a concentration of 5% for TT and TTN, respectively, but CL and CLN were not associated with significant larval mortality, even at high concentrations (Table 6). The LC_50_ values for TT and TTN were 3.11% and 3.55%, respectively, whereas they were much higher for CL (30.1%) and CLN (32.27%) (Table 7) (Figure 4).

### 3.6. Synergistic/Antagonistic Effects of Binary Mixtures of the Used Oils

In the adult immersion test, the TTCL combination resulted in a lower mortality rate than using TT or CL alone (Table 3). The synergistic ratio of 0.416 indicated that this combination had an antagonistic effect (Table 7, Figure 4). TTCL showed significant larval mortality even at low concentrations, with an LC_50_ value of 0.79%, indicating a synergistic effect (Table 7, Figure 4).

## 4. Discussion

The principal strategy used in tick control is the application of synthetic chemical acaricides. However, widespread and improper use of acaricides for tick control has resulted in significant resistance issues. Recently, there have been numerous reports on resistance to pyrethroid acaricides in ticks, for example, resistance to deltamethrin by *Rhipicephalus annulatus* [6]. Therefore, there is an urgent need to develop safe alternative strategies to effectively control tick populations, and EOs have been proposed as one such strategy [28].

In the current investigation, the application of TT and CL at all tested concentrations resulted in significant adult mortality (*p* < 0.05). However, CL only produced a significant ovicidal impact at high concentrations (10 and 5%), while TT had a non-significant ovicidal effect. The nanoemulsion forms of TTN and CLN showed non-significant mortality rates against adult ticks. At concentrations of 5% and 10%, TT and TTN showed considerable rates of larval death; meanwhile, CL and CLN did not appear to induce any larval deaths at any of the tested concentrations. Similar results were obtained by Pazinato et al. [12] who demonstrated 100% reproductive suppression and recorded a 70% acaricidal impact of TT and TT nanoparticles against *Rhipicephalus microplus* females. Additionally, TT oil showed effectiveness against *Ixodes ricinus* nymphs with the mortality rate reaching 80% at a concentration of 10 µL/mL [11]. Terpinene-4-ol, the primary constituent of TT oil, was shown to be the compound responsible for acaricidal activity [29]. 

Habeeb et al. [15] found significant toxic impacts of *Citrus limon* and *Citrus sinensis* against *Hyalomma dromedarii* ticks. Additionally, lemon oil was shown to be efficient for eradicating *Sarcoptes scabiei* mites in rabbits. Furthermore, treatment with citrus lemons significantly reduced the mosquito species’ larvae population [30,31] and produced significant insecticidal action against *Spodoptera frugiperda* [32] and *Aedes alopictus* larvae [33].

It is noteworthy that the current EO nanoemulsions displayed lower activity levels than the pure oils against adult ticks. Galli et al. [34] reported similar levels of effectiveness for pure, nanoemulsion, and nanocapsule forms of *Eucalyptus globulus* EO against *Rhipicephalus microplus* ticks. This result can be explained by the fact that a variety of factors, such as the temperature, storage time, pH, and ionic strength, have negative impacts on nanoemulsion stability. Furthermore, when an emulsion is kept at a certain temperature during its synthesis, the thermal energy of the droplets increases, raising the possibility of droplet collisions. Additionally, this lessens the viscosity of the interface, facilitating faster droplet coalescence and film drainage [35]. Although the EOs did not cause significant mortality rates in their nanoemulsion forms compared with their ordinary forms, they significantly reduced the reproductive efficacy of the treated ticks. In contrast, Dos Santos et al. [17] discovered that the nanostructured form of cinnamon essential oils had a high level of acaricidal effectiveness against *R*. (*B*.) *annulatus* ticks. In addition, Ibrahium et al. [20] found that a geranium oil nanoemulsion was more effective than the pure form against *R. annulatus*. The increased effectiveness of nanoemulsions over pure oils is a result of the smaller droplet size of EOs, which enhances the number of active molecule interactions with the biological target membranes and promotes their transfer through them [36]. Additionally, Gadelhaq et al. [37] showed that the D-limonene nanoemulsion maintains its efficacy for 2 months and has better efficacy than D-limonene in its unemulsified form. Furthermore, by lowering the LC_50_ to its half value, EO nanoemulsions showed enhanced effectiveness against *Aedes aegypti* larvae [38]. 

The insecticidal activity of an essential oil depends not only on its primary molecules but also on how all of its constituent parts interact with the physiology and behavior of an insect [39,40]. In addition to the essential oils’ toxicity, it is important to note that larvicidal effectiveness can be improved by using straightforward binary combinations [8]. The adulticidal activity level of the EO binary mix (TTCL), when a ratio of 1:1 was used, was less than those of the pure TT and CL oils alone. As a result, the combination had non-significant LC_50_ and LC_90_ values (4.93 and 12.1%, respectively) against adult ticks and an antagonistic effect with a synergistic factor of less than 1, although the TTCL was associated with high proportions of larval death at all concentrations. Additionally, the mixture had considerable LC_50_ and LC_90_ values (0.790 and 1.386%, respectively), demonstrating a synergistic impact with a synergistic factor greater than 1. Similarly, Benelli et al. [8] investigated binary combinations of various essential oils and discovered that *Pinus nigra* and *Aloysia citriodora* had a strong synergistic effect on *Culex quinquefasciatus* larvae. Meanwhile, they reported a low effectiveness for *Hyssopus officinalis* and *Pelargonium graveolens*. 

## 5. Conclusions

The pure TT and CL EOs had lower LC_50_ and LC_90_ values than the other two types (nanoemulsion and combination) against adult tick stages. The high CL concentrations (10 and 5%) showed the highest effective ovicidal activity levels. In contrast to their pure and nanoemulsion forms, the TT and CL binary mixture displayed potent larvicidal activity. Given that CL essential oil is affordable, further research into the use of CL on animals harmed by ticks needs to be undertaken.

## Figures and Tables

**Table 1:** GC-Mas of *tea tree* (TT) and *Citrus limon* (CL) oils.

RT	Compounds in TT	Area%	RT	Compounds in CL	Area%
3.30	Terpinene	52.24	4.54	trans-4-Thujanol	9.64
5.10	Dihydro-α-terpineol	5.97	4.87	alpha-terpineol	13.43
5.60	Diterpene	2.87	5.37	beta-Fenchyl alcohol	0.68
16.57	4-methyl-3-cyclohexen-1-yl	5.42	5.95	α- Terpinolene	5.81
24.02	Pentatriacontane	2.91	6.14	Dipropylene glycol	0.27
24.45	1-Octatriacontanol, pentafluoropropionate	4.20	6.61	Camphene hydrate	0.58
25.46	N-pentatriacontane	3.8	6.99	1-Terpinenol	0.56
25.86	17-Pentatriacontene	3.93	7.24	Menthol terpine hydrate	1.70
26.35	Octatriacontyl 2,2,3,3,3-pentafluoropropanoate	2.55	8.34	(L)-alpha-Terpineol	18.32
26.46	Tetrapentacontane, 1,54-dibromo-	3.31	9.16	cis-Citral	1.57
26.84	1,54-dibromotetrapentacontane	4.58	9.36	Linalool acetate	0.63
27.21	Octatriacontyl pentafluoropropionate	4.88	9.79	β-Geranial	2.21
28.16	Nonyl tetracosyl ether	3.35	11.4	α-Terpineol acetate	3.6
	Total	100	12.74	Diphenyl ether	24.56
			13.53	Widdrol hydroxyether	3.96
			13.65	methyl ester	2.76
			14.18	Citral propylene glycol acetal	5.73
			14.32	Geranial propylene glycol acetal	4.00
				Total	100

**Table 2:** Essential oil nanoemulsion characteristics.

Eos	TT	CL
The absorbance peak by UV-vis (nm)	270	345
Droplet size (d.nm)	188	50.66
PDI (d.nm)	0.527	0.171
Potential charges	−2.02	−2.30

PDI = polydispersity index.

**Table 3:** Adulticidal activity of different forms of *tea tree* (TT) and *citrus lemon* (CL) oils on *R. annulatus* (the ratio between oils binary mixture was 1:1) at 14 days post application.

Concentrations %	10	5	2.50	1.25	0.625
TT (DF %)	100.0 ± 00.00 *	96.66 ± 1.527 *	56.33 ± 8.020 *	41.00 ± 5.291 *	23.66 ± 3.785 *
TTN (DF %)	33.66 ± 2.081 *	17.00 ± 1.000	14.66 ± 0.1.527	5.666 ± 2.516	2.666 ± 1.527
CL (DF %)	100.0 ± 0.000 *	100.0 ± 0.000 *	80.66 ± 2.081 *	63.00 ± 4.358 *	36.33 ± 5.131 *
CLN (DF %)	37.33 ± 3.055	22.00 ± 3.000	13.33 ± 1.527	9.666 ± 0.577	3.666 ± 1.154
TTCL (DF %)	76.33 ± 0.577 *	53.00 ± 1.000 *	43.33 ± 2.081 *	35.66 ± 2.516 *	24.00 ± 2.645 *
Control (Deltamethrin 2 µL/mL)	0.000 ± 0.000				
Control (Phoxim 0.50 µL /mL)	100.0 ± 0.000				
Control (Ethyl alcohol 70%)	0.000 ± 0.000				

(*) significant compared with the control (Ethyl alcohol) (*p* < 0.05), DF % (female mortality %), TT (*tea tree* oil), TTN (*tea tree* nanoemulsion), CL (*citrus lemon* oil), CLN (*citrus lemon* nanoemulsion), TTCL (*tea tree* oil + Citrus limon oil).

**Table 4:** Egg production index (EPI) of different forms of TT and CL against *R. (B.) annulatus* at 14 days post application.

Concentrations %	10	5	2.50	1.25	0.625
TT (EPI %)	0.000 ± 0.000 *	3.163 ± 0.233 *	23.50 ± 1.600	25.10 ± 1.110	30.40 ± 0.088
TTN (EPI %)	23.16 ± 1.813	36.06 ± 2.674	43.72 ± 1.041	55.01 ± 2.602	65.33 ± 0.770
CL (EPI %)	0.000 ± 0.000 *	0.000 ± 0.000 *	8.263 ± 3.209 *	21.93 ± 0.474 *	28.09 ± 2.404
CLN (EPI %)	25.09 ± 1.127	30.39 ± 0.078	33.62 ± 3.298	47.48 ± 0.893	53.99 ± 1.923
TTCL (EPI %)	21.59 ± 0.923 *	24.49 ± 0.823	28.08 ± 2.055	30.25 ± 2.463	34.34 ± 1.006
Control (Deltamethrin 2 µL/1mL)	59.34 ± 7.987				
Control (Phoxim 0.50 µL/1mL)	0.000 ± 0.000				
Control (Ethyl alcohol 70%)	64.43 ± 7.623				

(*) significant compared with the control (Ethyl alcohol) (*p* < 0.05), DF % (female mortality %), TT (*tea tree* oil), TTN (*tea tree* nanoemulsion), CL (*citrus lemon* oil), CLN (*citrus lemon* nanoemulsion), TTCL (*tea tree* oil + Citrus limon oil).

**Table 5:** Ovicidal effect of different TT and CL EO forms and nanoemulsion forms.

Concentrations %	10	5	2.50	1.25	0.625
TT (Percentage of egg hatching inhibition mean ± SD)	13.66 ± 2.081	11.33 ± 2.516	8.566 ± 1.504	5.000 ± 1.000	2.333 ± 1.154
TTN (Percentage of egg hatching inhibition mean ± SD)	13.00 ± 2.000	10.66 ± 2.081	8.666 ± 3.055	5.666 ± 1.527	2.313 ± 1.527
CL (Percentage of egg hatching inhibition mean ± SD)	90.66 ± 3.055 *	69.00 ± 4.000 *	47.66 ± 3.055 *	5.666 ± 1.527	4.000 ± 1.000
CLN (Percentage of egg hatching inhibition mean ± SD)	69.00 ± 3.605 *	26.33 ± 3.511 *	15.00 ± 2.000	7.666 ± 1.527	4.666 ± 1.527
Control (Deltamethrin 2 µL/mL)	8.666 ± 1.527				
Control (Phoxim 0.50 µL/1mL)	82.66 ± 1.527				
Control (Ethyl alcohol 70%)	5.666 ± 2.082				

(*) significant compared with the control (Ethyl alcohol) (*p* < 0.05), TT (*tea tree* oil), CL (*Citrus limon* oil), TTN (*tea tree* nanoemulsion), CLN (*citrus lemon* nanoemulsion).

**Table 6:** Larvicidal effect of different forms of TT and CL (the ratio between oils in the 1:1 binary mixture) (mortality rate %).

Concentrations %	10	5	2.50	1.25	0.625
TT	100.0 ± 0.000 *	92.33 ± 2.516 *	22.00 ± 3.000	16.66 ± 3.785	11.66 ± 2.886
TTN	100.0 ± 0.000 *	83.33 ± 3.785 *	18.00 ± 4.582	11.33 ± 4.041	7.333 ± 2.516
CL	8.333 ± 3.511	7.000 ± 2.645	5.333 ± 1.527	4.333 ± 1.527	4.000 ± 1.000
CLN	8.666 ± 3.214	6.333 ± 2.516	4.666 ± 1.527	3.666 ± 1.527	3.333 ± 0.577
TTCL	100.0 ± 0.000 *	100.0 ± 0.000 *	100.0 ± 0.000 *	78.00 ± 2.645 *	47.66 ± 2.516 *
Control Deltamethrin (2 µL/1mL)	20.33 ± 0.577				
Control (Phoxim 0.50 µL/1mL)	100.0 ± 0.000				
Control (Ethyl alcohol 70%)	10.23 ± 0.432				

(*) significant compared with the control (Ethyl alcohol) (*p* < 0.05), TT (*tea tree* oil), TTN (*tea tree* nanoemulsion), CL (*citrus lemon* oil), CLN (*citrus lemon* nanoemulsion), TTCL (*tea tree* oil and *citrus lemon* oil).

**Table 7:** LC_50_, LC_90_, and synergistic factor for the adulticidal and larvicidal activity of TT and CL against *R. (B.) annulatus* (the ratio between oils in the 1:1 binary mixture).

Oil	Adulticidal Activity			Larvicidal Activity		
	LC_50_%	LC_90_%	Synergistic Factor	LC_50_%	LC_90_%	Synergistic Factor
TT	2.05	4.00		3.112	5.155	
TTN	12.3	20.8	0.167 (Antagonism)	3.554	5.705	0.875 (Antagonism)
CL	1.26	2.62		30.13	49.87	
CLN	11.4	19.9	0.110 (Antagonism)	32.27	53.67	0.933 (Antagonism)
TTCL	4.93	12.1	0.416 (Antagonism)	0.790	1.386	3.939 (Synergism)

TT (*tea tree* oil), TTN (*tea tree* nanoemulsion), CL (*citrus lemon* oil), CLN (*citrus lemon* nanoemulsion), TTCL (*tea tree* oil and *citrus lemon* oil).

## Data Availability

Not applicable.

## List of Abbreviations

TT (tea tree); TTN (*tea tree* nanoemulsion); CL (citrus lemon); CLN (citrus lemon nanoemulsion); *R. annulatus* (*Rhipicephalus annulatus*); EOs (essential oils); GC-MS (gas chromatography–mass spectrometry); PDI (polydispersity index); ALT (adult immersion test); LPT (larval packet test); EPI (egg production index); LC_50_ (lethal concentration of 50% of the population); LC_90_ (lethal concentration of 90% of the population).
